# Accurate Location in Dynamic Traffic Environment Using Semantic Information and Probabilistic Data Association

**DOI:** 10.3390/s22135042

**Published:** 2022-07-04

**Authors:** Kaixin Yang, Weiwei Zhang, Chuanchang Li, Xiaolan Wang

**Affiliations:** 1 School of Mechanical and Automotive Engineering, Shanghai University of Engineering Science, Shanghai 201620, China; ykx961028@163.com (K.Y.); zwwsues@163.com (W.Z.); xiaolanwang@sues.edu.cn (X.W.); 2 School of Vehicle and Mobility, Tsinghua University, Beijing 100084, China; 3 Shanghai Smart Vehicle Integration Innovation Center Co., Ltd., Shanghai 201620, China

**Keywords:** dynamic traffic environment, semantic information, probabilistic data association, Fast-SCNN

## Abstract

High-accurate and real-time localization is the fundamental and challenging task for autonomous driving in a dynamic traffic environment. This paper presents a coordinated positioning strategy that is composed of semantic information and probabilistic data association, which improves the accuracy of SLAM in dynamic traffic settings. First, the improved semantic segmentation network, building on Fast-SCNN, uses the Res2net module instead of the Bottleneck in the global feature extraction to further explore the multi-scale granular features. It achieves the balance between segmentation accuracy and inference speed, leading to consistent performance gains on the coordinated localization task of this paper. Second, a novel scene descriptor combining geometric, semantic, and distributional information is proposed. These descriptors are made up of significant features and their surroundings, which may be unique to a traffic scene, and are used to improve data association quality. Finally, a probabilistic data association is created to find the best estimate using a maximum measurement expectation model. This approach assigns semantic labels to landmarks observed in the environment and is used to correct false negatives in data association. We have evaluated our system with ORB-SLAM2 and DynaSLAM, the most advanced algorithms, to demonstrate its advantages. On the KITTI dataset, the results reveal that our approach outperforms other methods in dynamic traffic situations, especially in highly dynamic scenes, with sub-meter average accuracy.

## 1. Introduction

Real-time localization in the dynamic traffic environment is one of the essential technologies for unmanned autonomous vehicles (UAVs). The environment has high dynamic characteristics with many participants and significant scene changes. Simultaneous localization and mapping (SLAM) are often used to solve the problem of autonomous localization in unknown environments. It determines the current location of the autonomous vehicle based on the surrounding environment data observed by the sensors. The ability to deal with dynamic situations and changes, according to [1], is a significant problem for autonomous driving localization. Traditional SLAM systems make the assumption that all objects in the environment would remain static. These SLAM systems use outlier filtering approaches [2] and robust implicit penalties [3] to deal with dynamic environment difficulties, while Kerr et al. [4] show that these methods are only robust in low dynamic circumstances. The research of [5] also demonstrates that the topic of real-time dynamic environment localization is still unsolved and that the existing technical level needs to be improved further.

In recent years, deep learning has achieved great success in visual perception, and its inference speed and perception accuracy have achieved consistent performance improvements in autonomous driving applications. VSLAM can be combined with deep learning to jointly complete the real-time positioning task of autonomous vehicles. In this paper, the semantic information obtained by deep learning is added to the visual SLAM system, and a coordinated localization method of semantic information and probabilistic data association is proposed to meet the challenge of real-time localization in dynamic traffic environments. The improved semantic segmentation network extracts multi-scale granular features to understand better and describe scene semantic information. To ensure the quality of data association, semantic information is used to eliminate the interference of dynamic feature points. However, strictly removing interference may overlook matching pairings in the data connection to some extent. As a result, the expected measurement likelihood model is used in this work, which can identify the best estimate for the optimization model when the data is incomplete or contain unobserved latent data.

The main contributions of this paper can be summarized as follows:

(1) The improved semantic segmentation network, building on Fast-SCNN, uses the Res2net module instead of the Bottleneck in the global feature extraction to further explore the multi-scale granular features. It achieves the balance between segmentation accuracy and inference speed, leading to consistent performance gains on the coordinated localization task of this paper.
(2) The robust scene descriptor fuses geometric, semantic, and distributional information to improve the quality of data association.
(3) The probabilistic data association is created to find the best estimate using a maximum measurement expectation model. This approach assigns semantic labels to landmarks observed in the environment and is used to correct false negatives in data association.

## 2. Related Word

Astonishing progress has been made in SLAM technology, enabling large-scale applications and witnessing the development of autonomous positioning. SLAM technology can be divided into LIDAR SLAM and Visual SLAM according to different sensors. Since LIDAR is expensive, low-cost cameras are more suitable for commercial promotion, and visual SLAM has developed rapidly with computer vision in recent years. As early as 1999, P.M. Newman [6] studied vision and SLAM-related issues and confirmed that visual SLAM could learn from machine vision-related research results. People thought that only stereo cameras could achieve visual SLAM for a long time until A.J. Davison [7] used monocular cameras to complete SLAM, creating monocular visual SLAM. The PTAM framework, the basic framework of visual SLAM, was proposed by Klein G. and Murray D. [8], which comprises two threads of tracking and mapping. The Track thread uses FAST [9] to extract features and initially estimate the camera pose, and the Map thread uses the Bundle Adjustment [10] algorithm to correct the pose estimation deviation. Raúl Mur-Artal et al. proposed ORB-SLAM [11], which adds map initialization and closed-loop detection functions to the PTAM framework, optimizes keyframes selection and map construction and has good processing speed and map accuracy. The ORB-SLAM2 version [12] supports monocular, binocular, and RGB-D interfaces. Moreover, the ORB-SLAM3 version [13] adds IMU coupling and supports the fisheye camera, which can run stably in real-time in small and large indoor and outdoor scenes. These classic SLAM systems show outstanding performance in static or low-dynamic environments but cannot get rid of the interference of dynamic objects in high-dynamic environments.

Visual SLAM in dynamic environments has become a hot research topic. The systems can usually be divided into three methods to eliminate the effects of dynamic objects: direct, feature point, and deep learning. Alcantarilla P.F. et al. [14] used dense disparity maps and dense optical flow between consecutive frames to estimate dense 3D scene flow, which they paired with motion likelihood to detect moving objects. This method enhances localization and mapping outcomes for dense and dynamic situations by omitting erroneous measurement information from the estimation. Jiyu Chenga et al. [15] employed the optical flow of consecutive frames to differentiate dynamic feature points in an image. Dynamic feature points will be added to the feature map, and static feature points will be entered into the visual SLAM system to ensure the accuracy of the posture estimate. Forster C et al. [16] utilized a direct technique to monitor and triangulate high-gradient pixels and motion information and a robust probabilistic depth estimation algorithm to achieve greater accuracy in low-texture dynamic scenarios.

None of the above methods goes beyond traditional geometric reconstruction to improve the system’s understanding of the environment. With the rapid development of deep learning technology, more and more research attempts to introduce deep learning into SLAM, and some work has achieved good results. These jobs can be roughly divided into two categories. One category is deep learning methods to replace some modules in traditional visual SLAM [17,18,19,20]. The method for extracting depth information from picture pairs was proposed by Zbontar J. and LeCun Y. [17]. This method uses a convolutional neural network to learn picture similarity and a binary classification dataset for stereo matching to retrieve depth information. A lightweight point tracking system was devised by DeTone D. et al. [18]. In this system, a neural network extracts the image’s important 2D points, and another network predicts the homography of these points and matches them, boosting the tracking system’s real-time performance. Garg R. et al. [19] introduced an unsupervised convolutional neural network for single-view depth prediction, which addressed the shortcomings of manually annotated data. The network is comparable to other state-of-the-art slams in terms of performance. Borna Besic and Abhinav Valada [20] suggested an end-to-end deep learning architecture for filtering dynamic objects from RGB-D sequences and fixing occlusion regions in dynamic objects. Specifically, the generative adversarial network uses a gated loop feedback method to improve temporal consistency by training the model from coarse to fine. The model also adjusts the depth of the images, ensuring geometric consistency throughout the inpainting architecture’s end-to-end training.

Another study adds semantic information to classic SLAM technology by combining visual SLAM with deep learning [21,22,23,24]. DynaSLAM is a system proposed by Berta Bescos et al. [21] that uses deep learning and multi-view geometry to recognize dynamic objects, restore background frames, build static scene maps that reduce emotional interference, and improve localization performance in dynamic situations. DynaSLAM II, according to Berta Bescos et al. [22], combines instance semantic segmentation and ORB features with Object Data Association to add dynamic objects to Bundle Adjustment to monitor dynamic items. As a result, the environment around dynamic objects is better understood, and posture prediction is improved. DS-SLAM [23] combines a semantic segmentation network with motion consistency checking to decrease the influence of dynamic objects and generate dense semantic glyph maps. Yuxiang Sun et al. [24] used motion segmentation to optimize the loss function, resulting in more accurate results. Nikolay Atanasov et al. [25] employ object detection to extract semantic information from sensors, create maps with semantic labels, and solve the semantic localization problem using ensemble-based Bayesian filters in polynomial time.

Precision localization in autonomous driving scenarios has gotten a lot of interest from industry and research in recent years. Peiliang Li et al. [26] employed 2D boxes and viewpoint classification to construct lightweight 3D box inference systems. The rough initial position is immediately derived from the 2D frame in this work. The dynamic target tracking is completed utilizing the BA optimization method combined with semantic and features information. Wentao Cheng et al. [27] employed semantic information in the route to address the autonomous vehicle localization challenge. The CenterNet network is used to detect road semantic features, key points represent lane lines and road signs, and semantic associations are used to optimize the overall state. Tong Qin et al. [28] developed a lightweight autonomous driving positioning framework that included vehicle-side mapping, cloud-based maintenance, and user-side positioning. Learning-based semantic segmentation is used to extract significant landmarks. The semantic landmarks are then converted to 3D and registered on the local map. The cloud server will receive the local map. The data collected by different vehicles are combined by the cloud server, which compresses the global semantic map. Finally, for localization, the compact map is delivered to production vehicles.

In this paper, we examine the strengths and shortcomings of previous work and present a joint localization solution for dynamic traffic conditions. The technique makes heavy use of semantic priors and probabilistic data associations and a maximum expectation measurement estimation algorithm to achieve good pose estimate accuracy in the presence of unobserved latent data in varied dynamic traffic scenarios.

## 3. Method

### 3.1. System Overview

**Figure 1:**
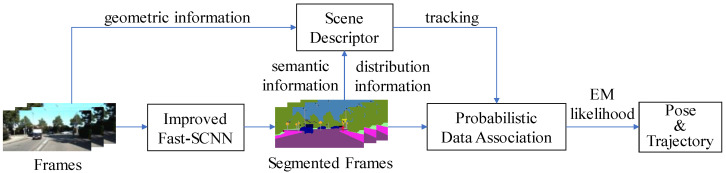
System overview, showing all the steps performed in semantic segmentation, scene description, and probabilistic data association.

Figure 1 depicts a high-level overview of the system framework. To accomplish pixel-level real-time semantic segmentation without losing accuracy, the video streams pass through an enhanced Fast-SCNN network. The system can swiftly delete dynamic feature points based on the semantic information received by the segmentation network to prevent impacting the quality of subsequent data linkages. In dynamic traffic conditions, more complex scene descriptors include geometric, semantic, and distributional information to increase localization accuracy. A maximum expectation measurement approach is used to predict the best camera posture and landmark locations by giving semantic labels to observed landmarks in the backdrop using probabilistic data association.

### 3.2. Dynamic Objects Segmentation and Culling

**Figure 2:**
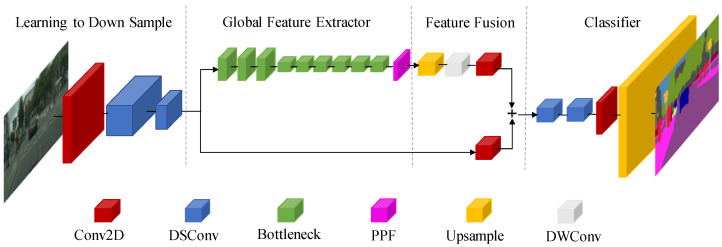
The Fast-SCNN network architecture consists of four parts: learning down sample, global feature extraction, feature fusion, and classifier. The network performs real-time semantic segmentation on the target in the camera frame and determines its dynamic and static attributes according to the category to which the pixel belongs.

**Figure 3:**
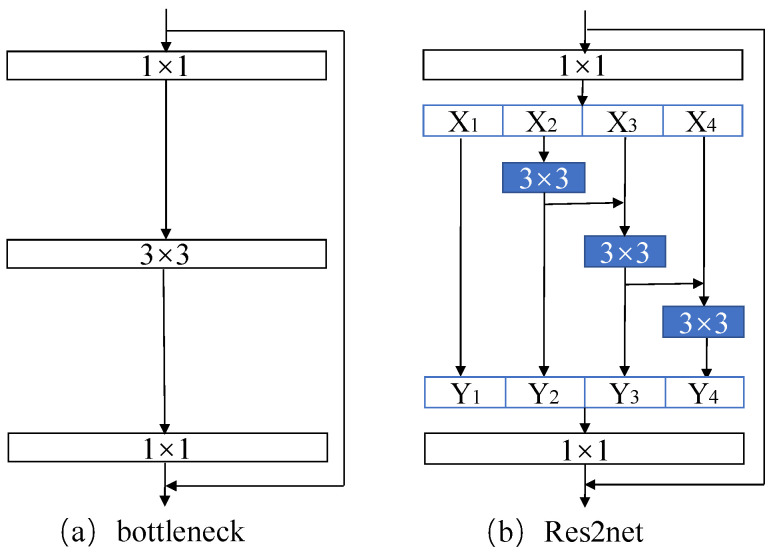
Compare the difference between (**a**) Bottleneck and (**b**) Res2net. Res2net represents more detailed multi-scale features, conducive to learning complex correlations between object categories and improving network prediction accuracy.

The fact that dynamic feature points participate in matching and contribute to localization failure is one of the most common visual SLAM system flaws. Fast-SCNN [29], a dual-branch encoder-decoder network, achieves pixel-wise segmentation of pixels in real-time, allowing dynamic objects to be quickly distinguished without compromising the SLAM system. At low resolution and full input resolution, Fast-deep SCNN’s and shallow two-layer networks collect global context and learn spatial features, respectively. The four modules of the Fast-SCNN network (shown in Figure 2) are all built using depth wise separable convolutions, which means they have less network parameters and faster segmentation, but they also have the problem of losing segmentation accuracy. This research offers the Res2net module [30] to replace the Bottleneck for multi-scale feature representation in order to address the problem that the global feature extraction of this network is rough, and that the segmentation impact is not perfect. The difference between Res2net and the Bottleneck block is shown in Figure 3.

The Res2net module creates hierarchical residual connections within the residual block, which may be put into the Fast-SCNN model’s backbone to achieve long-term performance improvements. The Res2net module separates the input feature maps into numerous groups, uses the previous group’s output map as the input for the next group, and then uses the 1 × 1 filter to fuse the feature maps of all groups. This module improves the receptive field of networks at all levels by extracting multi-scale features at the granularity level. It also effectively simplifies the complexity of the correlation between learning object categories and improves the accuracy of classification boundaries.

The modified Fast-SCNN network benefits from hierarchical residual connections, which improve segmentation accuracy without adding too many network parameters. The new network parameter is 1.27 million, which is only 0.16 million greater than the previous one. This ensures the network’s applicability in dynamic traffic conditions. The network’s performance is confirmed in experiment A, with segmentation accuracy 2.11 percent greater than the original version and inference speed 216.3 fps, striking a solid balance between inference speed and segmentation accuracy. Table 1 displays the segmented semantic labels, which include the majority of the object categories seen in vibrant traffic scenes.

### 3.3. Scene Description Using Geometric and Semantic Information

#### 3.3.1. Geometric Feature Descriptor

Given the real-time requirement of traffic scene localization, ORB [31] descriptor has the characteristics of rotation invariance and low computational cost and can quickly extract and match scene features. FAST corners are pulled on multiple scales of the Gaussian pyramid, and feature points at different levels are removed according to the allocation strategy of each layer. Equation (1) is the expression of the allocation strategy for each layer:

(1) $$N_{i}=\frac{N\left(1-s^{2}\right){\left({\left(s\right)}^{2}\right)}^{i}}{\left(1-{\left(s^{2}\right)}^{m}\right)}$$

where  N is the total number of extracted feature points, s is the scaling factor of the image pyramid, and m is the number of image pyramid layers.

The selection of feature points follows the principle: the pixel gray value changes beyond the threshold, and the semantic label in the corner of the static object. The following formula is used to express the selection criteria:

(2) $$m\left(x,y\right)=\omega_{ij}\sqrt{{\left(L\left(x+1,y\right)-L\left(x-1,y\right)\right)}^{2}+{\left(L\left(x,y+1\right)-L\left(x,y-1\right)\right)}^{2}}$$

(3) $$\theta \left(x,y\right)=\omega_{ij}tan^{-1}\left[\frac{L\left(x+1,y\right)-L\left(x-1,y\right)}{L\left(x,y+1\right)-L\left(x,y-1\right)}\right]$$

where $\omega_{ij}$ is the semantic label weight of the candidate feature point, the emotional type weight is set to 0, and the static target’s weight increases. The weights of the categories construction and object have been increased by three times, while the weights of the other categories have remained the same. The method is easy and effective for extracting static feature points, increasing the ratio of target feature points dispersed across construction and object categories, and boosting the quality of subsequent data association and ultimate positioning accuracy.

**Figure 4:**
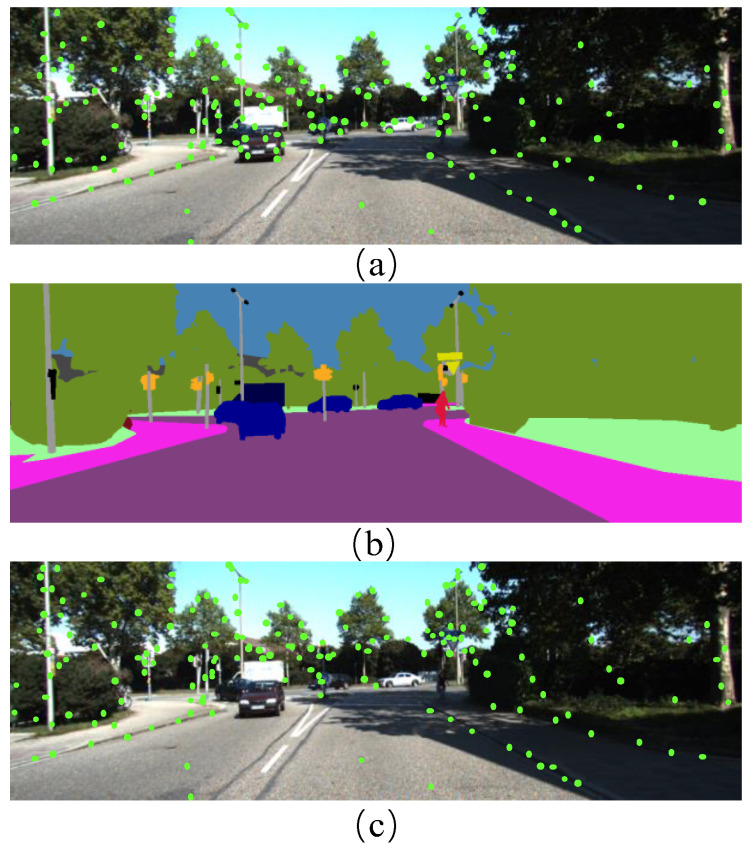
(**a**) Feature point map extracted from the original ORB descriptor; (**b**) Semantic segmentation renderings; (**c**) Feature point map for improved ORB descriptor extraction. After our improvement, the extracted feature points are no longer distributed on dynamic objects but more concentrated on objects with object categories such as poles and traffic lights.

The feature points are concentrated in the local part of the image, and the effect of the descriptor will be very unsatisfactory. To this end, the quadtree algorithm [32] can uniformize the feature points. The rendering of the final feature point extraction is shown in Figure 4. The ORB descriptor determines the orientation of feature points through intensity centroids and uses binary strings to describe the pixel variation information of feature points and their neighborhoods.

#### 3.3.2. Semantic Feature Descriptor

Descriptors based on visual geometric features cannot accurately describe dynamic traffic scenes due to visible aliasing or changes in visual appearance. Incorporating semantic information and distribution information into descriptors can improve the above problems. Descriptors that fuse semantic and geometric features tradeoff uniqueness and robustness. It is not affected by perspective transformation and can also solve the difficulty of matching multiple feature points with the same category between different frames.

The semantic segmentation network extracts high-level semantic information at different levels and becomes the original data for constructing semantic information. The improved Fast-SCNN above shows the competitiveness of pixel segmentation, and the following formula represents the extracted semantic information:

(4) $$S_{K}=\left(S_{k}^{c},S_{k}^{l},S_{k}^{s}\right)$$

where $S_{k}$ represents the semantic result of the kth segmentation, characterized by the category $S_{k}^{c}$, the position $S_{k}^{l},$ and the confidence $S_{k}^{s}$ of the pixel point.

**Figure 5:**
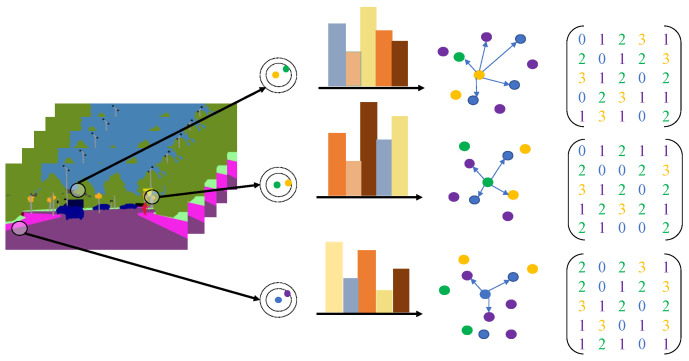
Our proposed semantic descriptor is based on semantic information and its distribution. The semantic segmentation network extracts high-level semantic information at different levels and selects the feature points with the most significant changes in semantic information as key points. The semantic information distribution of its neighborhood is analyzed for key points, and the semantic descriptors are represented in matrix form.

Static objects commonly found in traffic environments can provide robust descriptive features, so semantic context descriptors with inherently static features are generated in this work. The points with dramatic changes in semantics, that is, the feature points where the category of pixel points changes, are selected as the key points of semantic information description, and Equation (5) is used to express the selection of key points. The construction of the semantic descriptor is to aggregate the features of key points and the distribution features from the neighborhood and tell them in the form of a matrix. Figure 5 shows the construction process of the semantic descriptor.

(5) $$S_{D}\left(P,P^{\prime }\right)=\sum sgn\left(P_{\left(m,n\right)}-P_{\left(m,n\right)}^{\prime }\right)\;\;P,P^{\prime }\in S_{k\left(m,n\right)}^{c}$$

where sgn() is a sign function. When the pixel types in the semantic descriptor of the key point are the same, it is recorded as 1. Otherwise, it is 0.

### 3.4. Probabilistic Data Association

**Figure 6:**
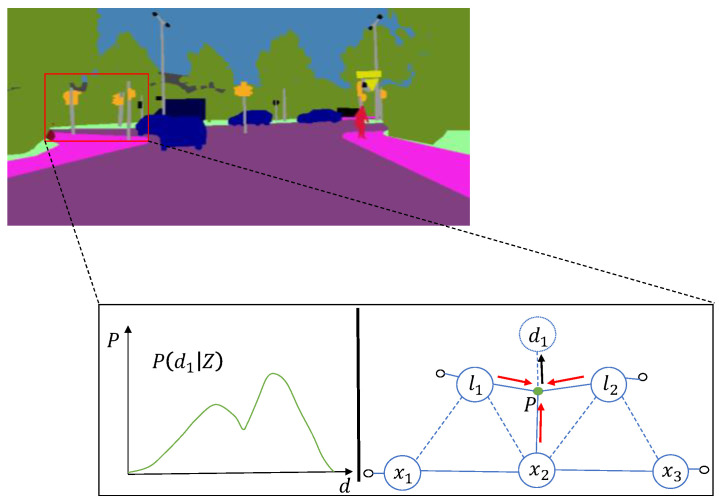
The illustrative overview of our proposed probabilistic data association approach. Top: Semantically segmented multiple objects with the same semantic label, and these objects are occluded. Bottom: Finding the optimal combination based on the current expected measurement likelihood model.

Data association aims to establish a mapping of sensor observations ${\left\{Z_{t}\right\}}_{t=1}^{T}$ to road sign positions ${\left\{l_{m}\right\}}_{m=1}^{M}$ and vehicle attitude ${\left\{X_{t}\right\}}_{t=1}^{T}$ relation. The traditional SLAM pose estimation optimization is divided into two steps, firstly estimating the data association, and then substituting the data association estimation results into the pose and road sign estimation. This leads to data association results that greatly affect the accuracy of pose estimation optimization. To this end, probabilistic data association methods add semantic labels to observed landmarks in the environment, improving the problem of data association accuracy. Figure 6 is an illustrative overview of a probabilistic data association method. The maximum expected measurement likelihood model [33] considers the overall distribution of data associations and poses estimation as an overall optimization problem. This method finds the maximum estimate for an optimized model when data associations are incomplete or when there are unobserved latent data. The overall optimization model is specifically expressed as:

(6) $$X^{i+1},L^{i+1}=\arg\underset{X,L}{\max}E_{D}\left[\log P(Z|X,L,D)|X^{i},L^{i},Z\right]$$

where $X^{i},\;L^{i}$ represent the initial sensor attitude and road sign estimations, respectively. $E_{D}$ represents all the predicted values associated with the data, which can be warped as:

(7) $$X^{i+1},L^{i+1}=\arg\underset{X,L}{\max}\underset{D\in \hat{D}}{\sum }P\left(D|X^{i},L^{i},Z\right)\log P(Z|X,L,D)$$

The estimated value will change drastically with the camera pose, landmark position, and landmark category, traverse the possibilities of all data associations under $X^{i},L^{i}$, and Z until an optimal global value maximizes the overall weight. The expected and observed values are obtained from the specific data-related expected value and the observed value corresponding to the overall expected maximum value. At this time, these values are the optimal solution combination for the system. The above Equation (7) can be transformed:

(8) $$X^{i+1},L^{i+1}=\arg\underset{X,L}{\max}\overset{K}{\underset{k=1}{\sum }}\overset{n}{\underset{j=1}{\sum }}\omega_{k_{j}}\log P(z_{k}|x_{\alpha k},l_{\beta k})$$

where $\omega_{k_{j}}$ is the data correlation value corresponding to the overall expected maximum value, and $x_{\alpha k},\;l_{\beta k}$ are the equivalent sensor observation values at this moment.

## 4. Experiments

Experiments are performed on this scheme on the KITTI dataset [34] to test its performance in dynamic traffic environments. All investigations are implemented on Ubuntu18.04, NVIDIA-Linux-x_64-460.84, and CUDA11.1 development tools. The improved Fast-SCNN network is implemented on the PyTorch deep learning framework using Python, and the rest is implemented in C++ on the ROS operating system [35].

### 4.1. Evaluation of Models for Extracting Semantic Information

The upgraded Fast-SCNN network extracts picture semantic information, which is used to eliminate dynamic feature points and build semantic descriptors. The original Fast-SCNN network implementation is used in the trials, and the Bottleneck module is replaced by Res2Net to improve semantic segmentation accuracy. On two NVIDIA TITAN Xp GPUs, the network is trained with Batch Size 256 mini-batches. With an initial value of 0.001, the learning rate is dynamically set. The momentum coefficient was initially fixed to 0.5, but over several epochs, it was gradually annealed to 0.9.

This research compares the performance of the upgraded Fast-SCNN network and the original grid on the KITTI semantic segmentation dataset [36] to demonstrate its effectiveness. All experiments are conducted on laboratory workstations developed with the PyTorch deep learning framework to maintain constant experimental circumstances. IoU Class, IoU Category, and FPS are used to assess the model’s performance.

(9) $$\mathrm{IoU}=\frac{TP}{TP+FP+FN}$$

where TP represents the number of positive examples predicted by the model and the actual number of positive samples, FP represents the number of negative examples predicted by the model but positive examples, and FN represents the number of negative examples predicted by the model but positive examples. The calculation formulas of the IoU Class and IoU Category are similar, and the difference is that the objects are different.

It can be seen from Table 2 that the performance of the improved method in the IoU Class and IoU Category is 1.25% and 2.11% higher than the original method, respectively. Although the processing speed is slightly inferior to the original network, it also meets the real-time segmentation requirements in dynamic traffic scenarios. It achieves an effective balance between inference speed and segmentation accuracy.

### 4.2. Evaluation of Positioning Accuracy under KITTI Dataset

In the experiments, the image pyramid is set to m = 8, s = 1/1.2. In the feature point extraction stage, FAST-9 is chosen, and the threshold is set low enough to obtain more corner points. Harris corner filter selects appropriate corners as feature points. When generating the semantic descriptor, its size is specified to 21 × 21 and the threshold to 55, which has the best performance.

The localization performance is comprehensively evaluated on the KITTI dataset to verify the effectiveness and superiority of the association of scene descriptors and probabilistic data [25]. The KITTI odometer benchmark consists of 22 stereo sequences containing real-world data collected in urban, rural, and highway scenes. According to the degree of scene dynamics, comparative experiments are performed on static sequences (KITTI 00), low dynamic sequences (KITTI 04, 05), and high dynamic sequences (KITTI 01, 09).

Considering the errors caused by other factors unrelated to the algorithm, the evaluation indicators include relative pose error (RPE) and absolute trajectory error (ATE). RPE is used to evaluate the system’s anti-drift performance, while ATE assesses the system’s comprehensive positioning capability. The findings of the root mean square error (RMSE) comparison between systems are shown in Table 3. The less the root mean square error, the more accurate the posture estimation is and the better the system’s overall performance is. The best accuracy is indicated in black. It can be seen from the table that compared with other systems, this scheme has the best performance in all dynamic sequences; especially in high dynamic sequences, the performance is greatly improved. The effect is very similar to the state-of-the-art ORB-SLAM2 system in static sequences.

**Figure 7:**
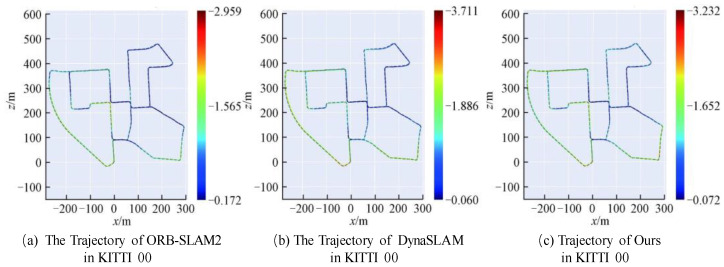
Comparison of camera translation trajectory errors in static sequence. (**a**–**c**) are the performance of ORB-SLAM2, DynaSLAM and our method on KITTI 00, respectively. Blue indicates the smallest trajectory error, followed by green, and red indicates the largest trajectory error.

The tracking trajectories in 3D space are converted to 2D space and plotted in the same graph as the ground truth to express the experimental comparison results more intuitively. The performance of the three algorithms in the static sequence (KITTI 00) environment is not much different, as shown by the visualization results of the camera trajectory error in Figure 7. The estimated value of the camera trajectory is not much different from the ground truth, and they are all relatively precise.

**Figure 8:**
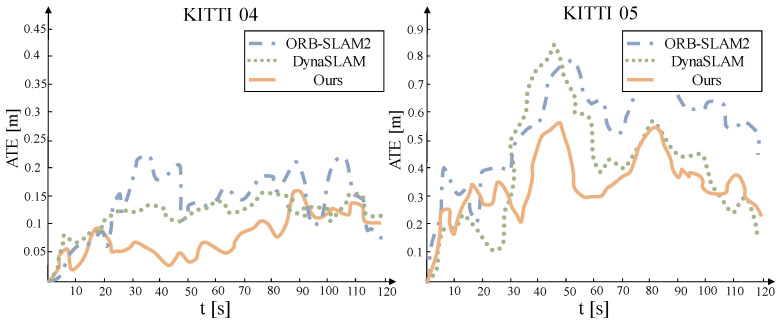
Comparison of camera translation trajectory errors in low-dynamic sequence.

The absolute trajectory error of the system under a low dynamic sequence is shown in Figure 8. This method can overcome the interference of dynamic objects and has the best performance in ground truth trajectory estimation. However, this advantage is not prominent in low dynamic scenarios. The reason may be due to the (RANSAC) outlier detection method used by ORB-SLAM2 and its resistance to a certain degree of active interference.

**Figure 9:**
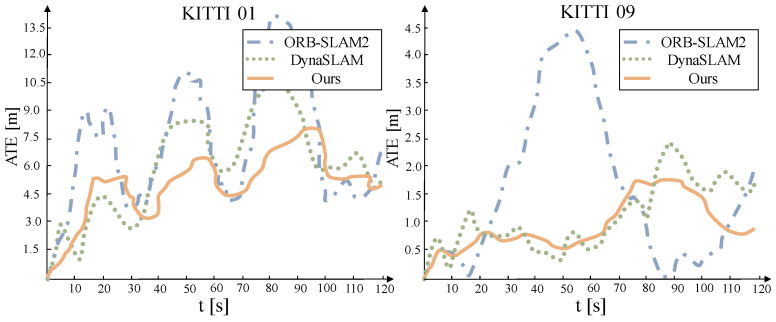
Comparison of camera translation trajectory errors in high-dynamic sequence.

As shown in Figure 9, in sequences with high dynamics and large scene changes, the outlier detection method used by ORB-SLAM2 is no longer applicable. It is affected by dynamic objects, and its estimated camera trajectory has a large difference from the ground truth, and even serious errors in some places. At the same time, the performance of this system is far superior to that of DynaSLAM. Although the system’s accuracy is slightly lower than DynaSLAM at certain moments, the error is quickly fixed, and the RMSE decline is significantly smaller than DynaSLAM. This could be due to the difficulty of matching the characteristics of low-texture regions with too comparable scenes. The maximum expected measurement estimation model could predict the system’s excellent pose value in the case of insufficient data association. 

## 5. Conclusions

For changing traffic settings, research-based on-scene descriptors and probabilistic data association give precise localization solutions. The new Fast-SCNN network recovers multi-scale features at a higher granularity level and extracts semantic information more quickly without sacrificing spatial information. The approach overcomes the negative impacts of dynamic targets on a broad scale using semantic information and prior knowledge. More complex scene descriptors aggregate geometric information, semantic information, and distribution information, which improves the accuracy of feature point matching. When there is unobserved potential data, the probabilistic data association approach finds the best-estimated value for the optimization model to achieve accurate positioning in dynamic traffic circumstances.

Comparative experiments with other excellent SLAM systems show that this method can achieve the highest accuracy in high and low dynamic traffic scenes. Although this research has made some progress in robustness and accuracy, there is still a long way to go. On the one hand, follow-up work strengthens research on precise localization in dynamic traffic environments with significant visual changes. It increases the applicability of SLAM systems in more challenging scenarios. On the other hand, the technology will be tested and fine-tuned in real traffic environments to improve the system’s ability to handle dynamic objects.

## Figures and Tables

**Table 1:** Categories of semantic segmentation.

Categories	Properties	Detail
human	dynamic	person, rider
vehicle	dynamic	car, truck, bus, bicycle, motorcycle, on-rails
animal	dynamic	dog, cat, bird, deer
construction	static	building, wall, guard-rail, fence, bridge, tunnel
object	static	pole, traffic-sign, traffic-light
flat	static	road, sidewalk, ground, parking, rail track
nature	static	vegetation, terrain

**Table 2:** Comparing the performance of our proposed improved Fast-SCNN network and the original network on the KITTI semantic segmentation test set.

Model	IoU Class (%)	IoU Category (%)	Input Size	FPS (fps)
Fast-SCNN	62.64	84.17	1242 × 375	265.8
Ours	63.89	86.28	1242 × 375	216.3

**Table 3:** Comparison of camera translation trajectory errors under KITTI datasets.

Sequence	ORB-SLAM2			DynaSLAM			Ours		
	RPE %	RPE °/100 m	ATE m	RPE %	RPE °/100 m	ATE m	RPE %	RPE °/100 m	ATE m
KITTI 00	**0.7**	**0.25**	**1.3**	0.74	0.26	1.4	0.71	**0.25**	1.37
KITTI 01	1.39	0.21	10.4	1.57	0.22	9.4	**1.15**	**0.16**	**8.80**
KITTI 02	0.76	**0.23**	5.7	0.80	0.24	6.7	**0.69**	0.24	**5.54**
KITTI 03	0.71	0.18	**0.6**	**0.69**	0.18	**0.6**	**0.69**	0.18	0.63
KITTI 04	0.48	0.13	0.2	0.45	**0.09**	0.21	**0.43**	0.11	**0.19**
KITTI 05	0.40	0.16	0.8	0.40	0.16	0.8	**0.37**	0.16	**0.68**
KITTI 06	0.51	**0.15**	0.8	0.50	0.17	0.8	**0.48**	**0.15**	0.80
KITTI 07	**0.50**	0.28	0.5	0.52	0.29	0.5	**0.50**	**0.25**	**0.44**
KITTI 08	**1.05**	0.32	3.6	**1.05**	0.32	3.5	1.07	0.32	**3.49**
KITTI 09	**0.87**	0.27	3.2	0.93	0.29	1.6	0.88	**0.26**	**1.47**
KITTI 10	0.60	**0.27**	**1.0**	0.67	0.32	1.2	**0.58**	**0.27**	1.02

## Data Availability

Not applicable.

## References

[1] Bresson G., Alsayed Z., Yu L., Glaser S. (2017). Simultaneous localization and mapping: A survey of current trends in autonomous driving. IEEE Trans. Intell. Veh..

[2] Vidal F.S., Barcelos A.D.O.P., Rosa P.F.F. Slam solution based on particle filter with outliers filtering in dynamic environments. Proceedings of the 2015 IEEE 24th International Symposium on Industrial Electronics.

[3] Gutiérrez-Gómez D., Mayol-Cuevas W., Guerrero J.J. Inverse depth for accurate photometric and geometric error minimisation in RGB-D dense visual odometry. Proceedings of the 2015 IEEE International Conference on Robotics and Automation.

[4] Kerl C., Sturm J., Cremers D. Robust odometry estimation for RGB-D cameras. Proceedings of the 2013 IEEE International Conference on Robotics and Automation.

[5] Singandhupe A., La H.M. A review of slam techniques and security in autonomous driving. Proceedings of the 2019 3th IEEE International Conference on Robotic Computing.

[6] Newman P.M. (1999). On the Structure and Solution of the Simultaneous Localization and Map Building Problem. Ph.D. Thesis.

[7] Davison A.J., Reid I.D., Molton N.D., Stasse O. (2007). MonoSLAM: Real-time single camera SLAM. IEEE Trans. Pattern Anal. Mach. Intell..

[8] Klein G., Murray D. Parallel tracking and mapping for small AR workspaces. Proceedings of the 2007 6th IEEE and ACM International Symposium on Mixed and Augmented Reality.

[9] Förstner W., Gülch E. A fast operator for detection and precise location of distinct points, corners and centres of circular features. Proceedings of the ISPRS Intercommission Conference on Fast Processing of Photogrammetric Data.

[10] Triggs B., McLauchlan P.F., Hartley R.I., Fitzgibbon A.W. Bundle Adjustment—A Modern Synthesis. Proceedings of the International Workshop on Vision Algorithms.

[11] Mur-Artal R., Montiel J.M.M., Tardos J.D. (2015). ORB-SLAM: A versatile and accurate monocular SLAM system. IEEE Trans. Rob..

[12] Mur-Artal R., Tardós J.D. (2017). ORB-SLAM2: An open-source SLAM system for monocular, stereo, and RGB-D cameras. IEEE Trans. Rob..

[13] Campos C., Elvira R., Rodríguez J.J.G., Montiel J.M., Tardós J.D. (2021). ORB-SLAM3: An accurate open-source library for visual, visual-inertial and multi-map SLAM. IEEE Trans. Rob..

[14] Alcantarilla P.F., Yebes J.J., Almazán J., Bergasa L.M. On combining visual SLAM and dense scene flow to increase the robustness of localization and mapping in dynamic environments. Proceedings of the 2012 IEEE International Conference on Robotics and Automation.

[15] Cheng J., Sun Y., Meng M.Q.H. (2019). Improving monocular visual SLAM in dynamic environments: An optical-flow-based approach. Adv. Rob..

[16] Garg R., Bg V.K., Carneiro G., Reid I. (2016). SVO: Semidirect visual odometry for monocular and multicamera systems. IEEE Trans. Rob..

[17] Zbontar J., LeCun Y. (2016). Stereo matching by training a convolutional neural network to compare image patches. J. Mach. Learn. Res..

[18] DeTone D., Malisiewicz T., Rabinovich A. (2017). Toward geometric deep slam. arXiv.

[19] Garg R., Bg V.K., Carneiro G., Reid I. Unsupervised cnn for single view depth estimation: Geometry to the rescue. Proceedings of the ECCV 2016: European Conference on Computer Vision.

[20] Besic B., Valada A. (2022). Dynamic object removal and spatio-temporal RGB-D inpainting via geometry-aware adversarial learning. IEEE Trans. Intell. Veh..

[21] Bescos B., Fácil J.M., Civera J., Neira J. (2018). DynaSLAM: Tracking, Mapping and Inpainting in Dynamic Scenes. IEEE Robot. Autom. Lett..

[22] Bescos B., Campos C., Tardós J.D., Neira J. (2021). DynaSLAM II: Tightly-coupled multi-object tracking and SLAM. IEEE Rob. Auto. Lett..

[23] Yu C., Liu Z., Liu X.J., Xie F., Yang Y., Wei Q., Fei Q. DS-SLAM: A semantic visual SLAM towards dynamic environments. Proceedings of the 2018 IEEE/RSJ International Conference on Intelligent Robots and Systems (IROS).

[24] Sun Y., Liu M., Meng M.Q.H. (2017). Improving RGB-D SLAM in dynamic environments: A motion removal approach. Rob. Auton. Syst..

[25] Atanasov N., Zhu M., Daniilidis K., Pappas G.J. Semantic Localization Via the Matrix Permanent. Proceedings of the Robotics: Science and Systems 2014.

[26] Li P., Qin T. Stereo vision-based semantic 3d object and ego-motion tracking for autonomous driving. Proceedings of the 2018 European Conference on Computer Vision (ECCV).

[27] Cheng W., Yang S., Zhou M., Liu Z., Chen Y., Li M. (2021). Road Mapping and Localization Using Sparse Semantic Visual Features. IEEE Rob. Autom. Lett..

[28] Qin T., Zheng Y., Chen T., Chen Y., Su Q. A Light-Weight Semantic Map for Visual Localization towards Autonomous Driving. Proceedings of the 2021 IEEE International Conference on Robotics and Automation (ICRA).

[29] Poudel R.P., Liwicki S., Cipolla R. (2019). Fast-SCNN: Fast semantic segmentation network. arXiv.

[30] Gao S.H., Heng M.M., Zhao K., Zhang X.Y., Yang M.H., Torr P. (2019). Res2net: A new multi-scale backbone architecture. IEEE Trans. Pattern Anal. Mach. Int..

[31] Rublee E., Rabaud V., Konolige K., Bradski G. ORB: An efficient alternative to SIFT or SURF. Proceedings of the 2011 International Conference on Computer Vision.

[32] Shaffer C.A., Samet H. (1987). Optimal quadtree construction algorithms. Comput. Vis. Graph. Image Process..

[33] Bowman S.L., Atanasov N., Daniilidis K., Pappas G.J. Probabilistic data association for semantic slam. Proceedings of the 2017 IEEE International Conference on Robotics and Automation.

[34] Geiger A., Lenz P., Stiller C., Urtasun R. (2013). Vision meets robotics: The kitti dataset. Int. J. Rob. Res..

[35] Quigley M., Conley K., Gerkey B., Faust J., Foote T., Leibs J., Ng A.Y. ROS: An open-source Robot Operating System. Proceedings of the ICRA Workshop on Open Source Software.

[36] Abu Alhaija H., Mustikovela S.K., Mescheder L., Geiger A., Rother C. (2018). Augmented reality meets computer vision: Efficient data generation for urban driving scenes. Int. J. Comput. Vis..

